# Supplementary material for: Novel Genetic Markers for Early Detection of Elevated Breast Cancer Risk in Women
Source: Int J Mol Sci. 2019 Sep 28;20(19):4828. doi: 10.3390/ijms20194828 (PMC6801521; doi:10.3390/ijms20194828)
Supplement: Supplementary file 1 [file ijms-20-04828-s001.pdf]

Supplementary Material

Novel genetic markers for early detection of elevated breast cancer risk in women
Bohua Wu, Yunhui Peng, Julia Eggert and Emil Alexov

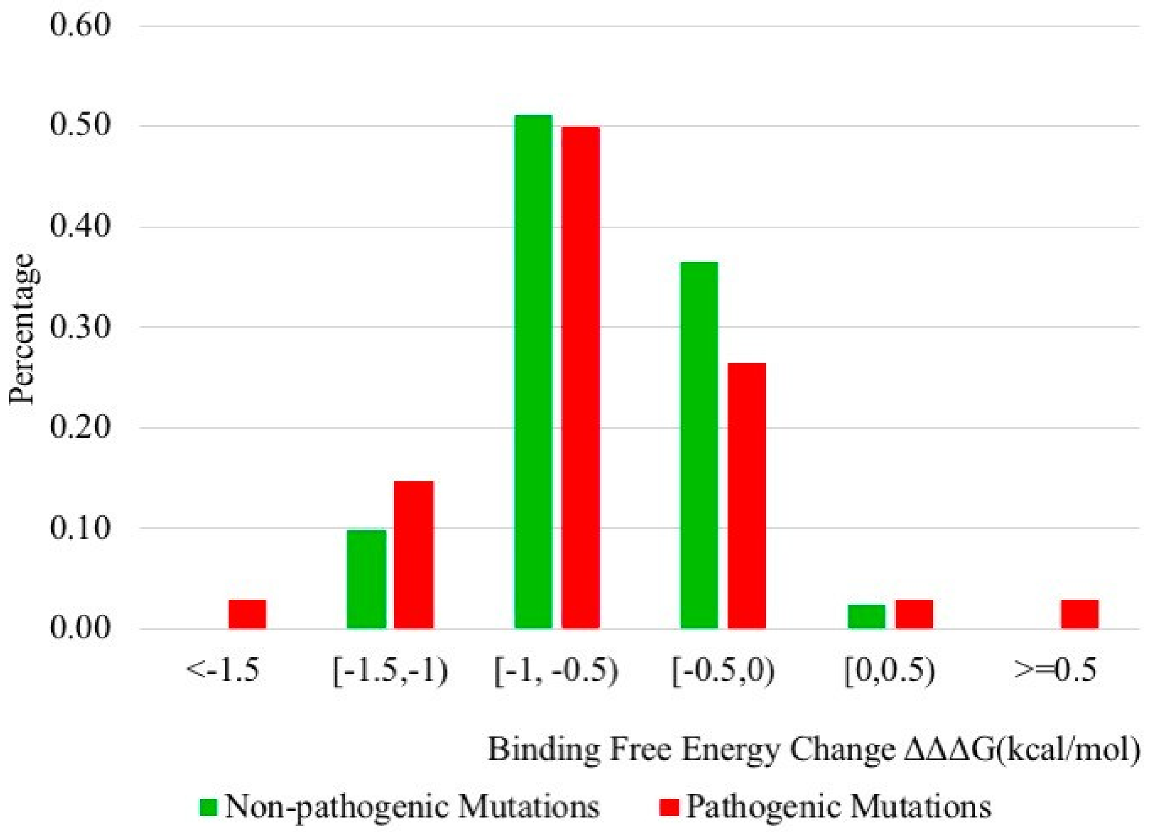

Figure S1. Binding Free Energy Change upon *MSH2* Missense Mutations. The binding free energy is not able to discriminate pathogenic missense mutations from those that are benign. Probably the reason is that the mutations are not located on the protein-protein interface when mapped onto the 3D structure.

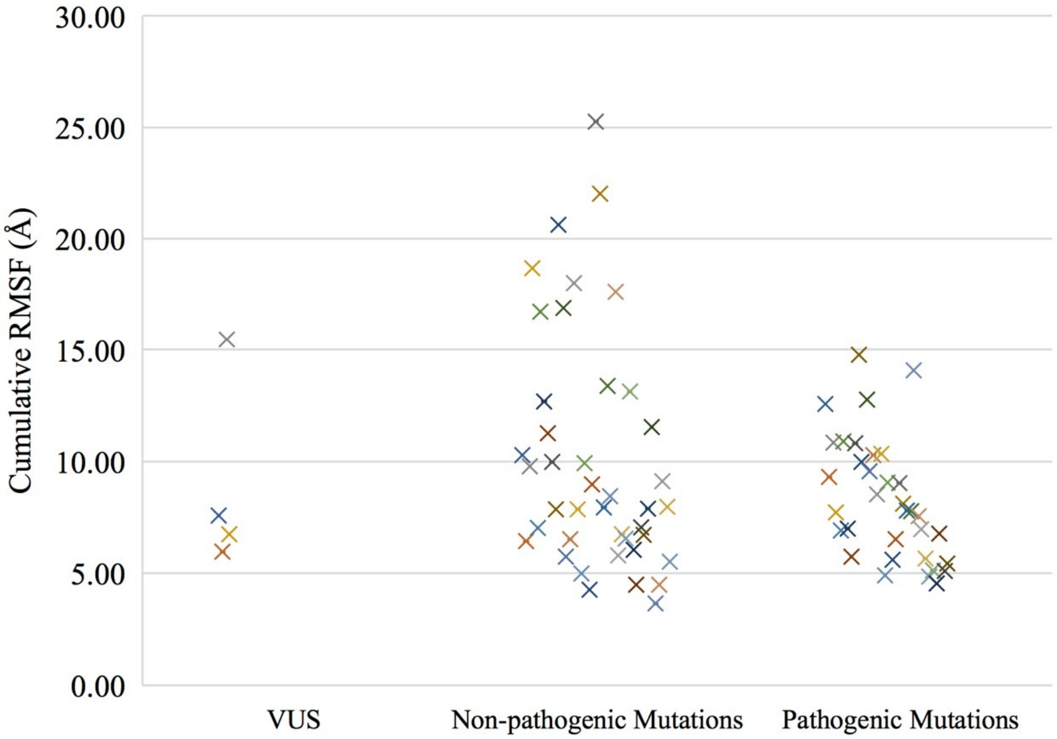

Figure S2A. Cumulative RMSF of 11 Neighborhood Residues Around the MSH2 Mutation. Some mutations have very big RMSF indicating that the neighborhood is more flexible. Each cross marker present one mutation data, and no relationship between the colors. Each cross present one data and no relationships between the colors.

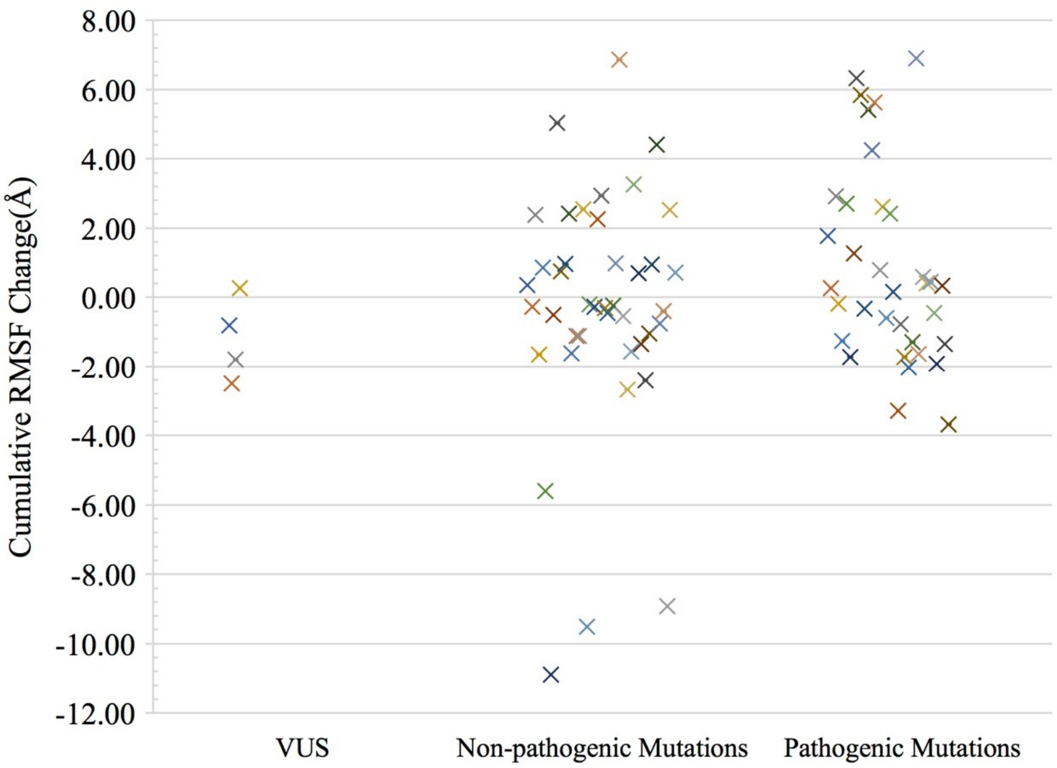

Figure S2B. Cumulative RMSF Change of 11 Neighborhood Residues upon *MSH2* Mutation. Cumulative RMSF Change with some mutations are large, however, it is not significant between pathogenic group and non-pathogenic group. Each cross present one data and no relationships between the colors.

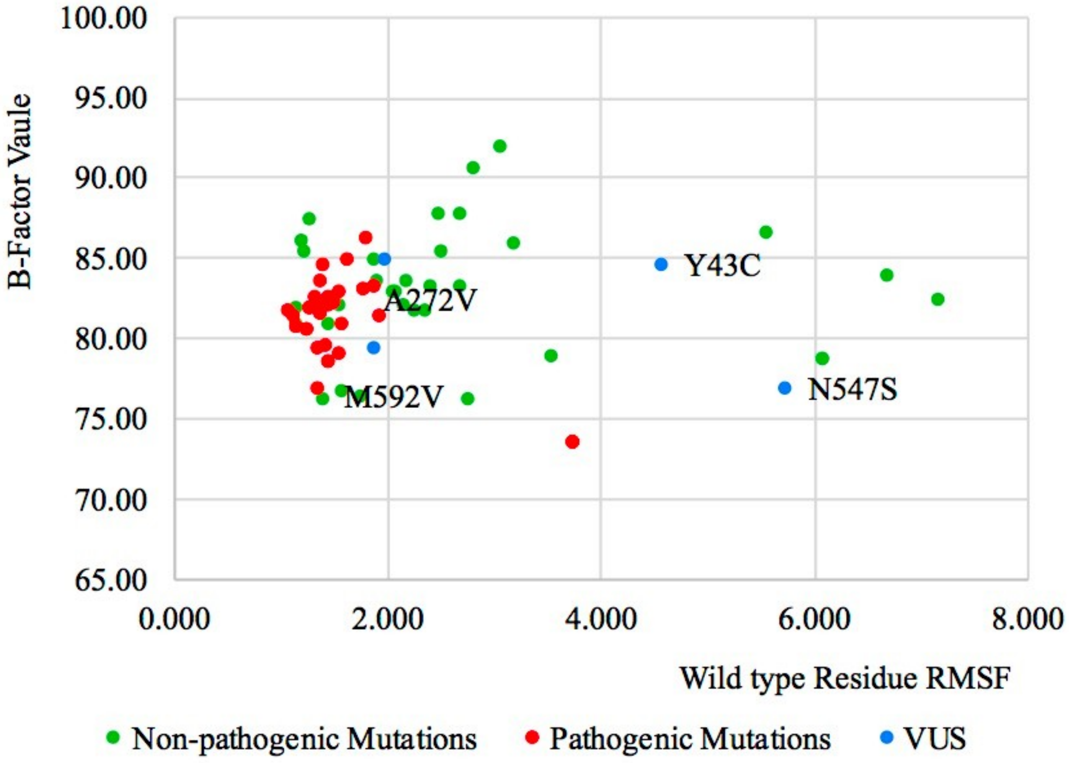

Figure S3. The Distribution of B-Factors and the RMSF of Wild-Type Residue on MSH2 Protein. Pathogenic locations have a smaller RMSF and smaller B-factor compared to non-pathogenic mutation locations.

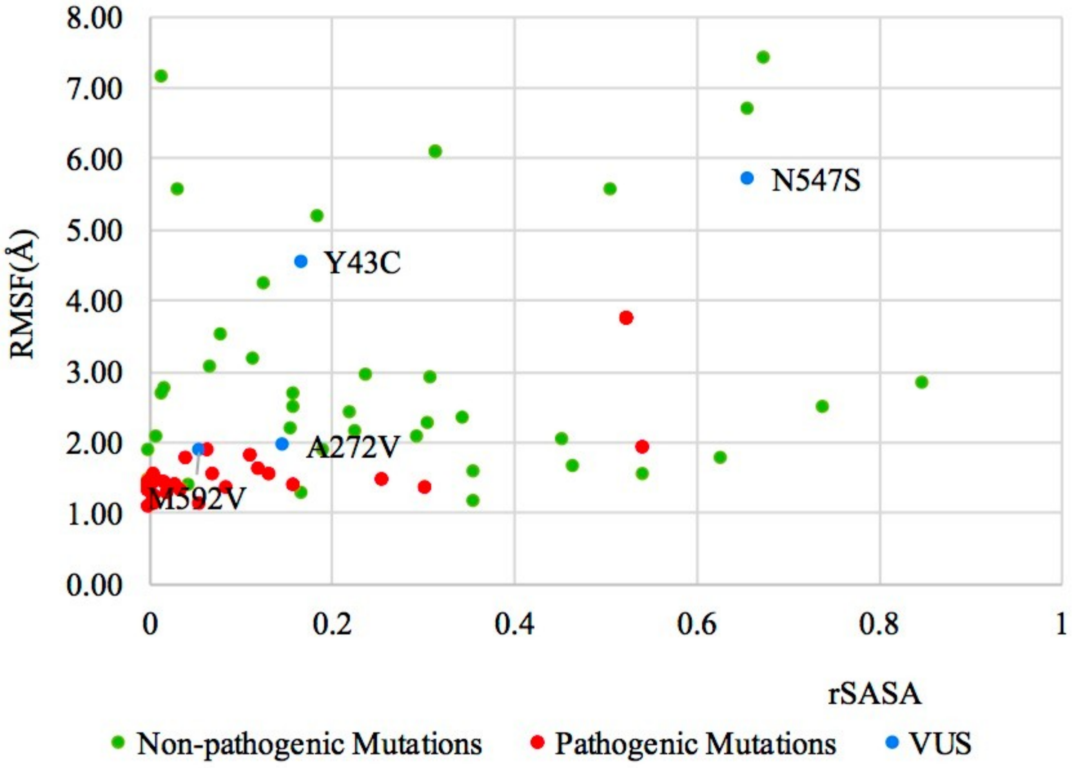

Figure S4. The Distribution of RMSF and rSASA Values of Wild-Type Residues on MSH2 Protein. Pathogenic locations have a smaller RMSF and smaller rSASA compared to non-pathogenic mutation locations.

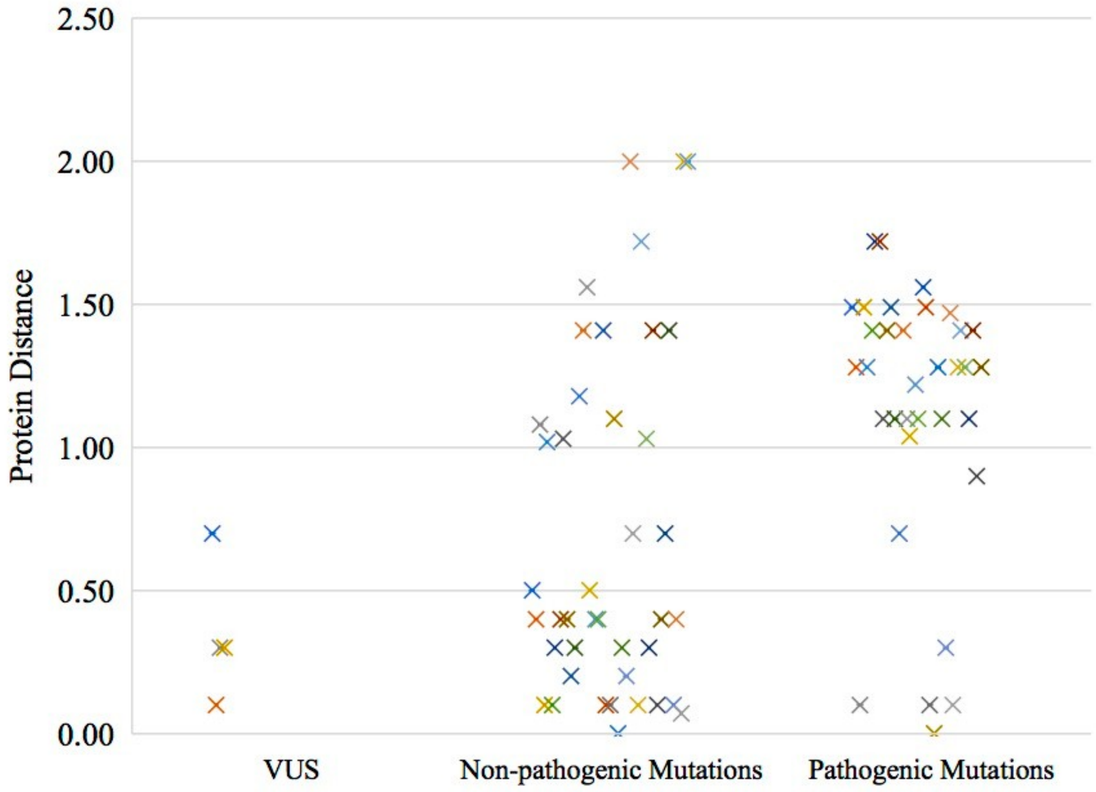

Figure S5. Protein Distance for VUS, Non-Pathogenic and Pathogenic MSH2 Mutations. The protein distance value upon mutation is smaller in most mutations in the non-pathogenic group than the pathogenic group. Each cross present one data and no relationships between the colors.

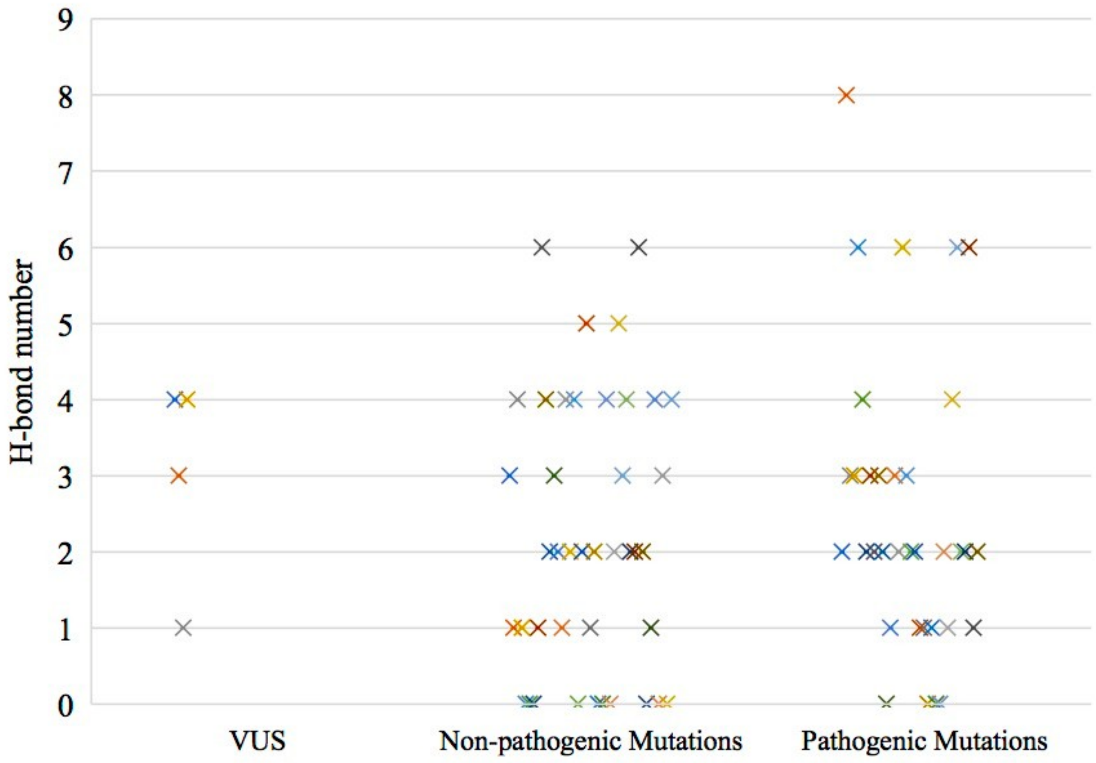

Figure S6A. H-Bond Numbers on MSH2 Mutant Residues. No significant difference between Non-pathogenic and pathogenic groups. Each cross present one data and no relationships between the colors.

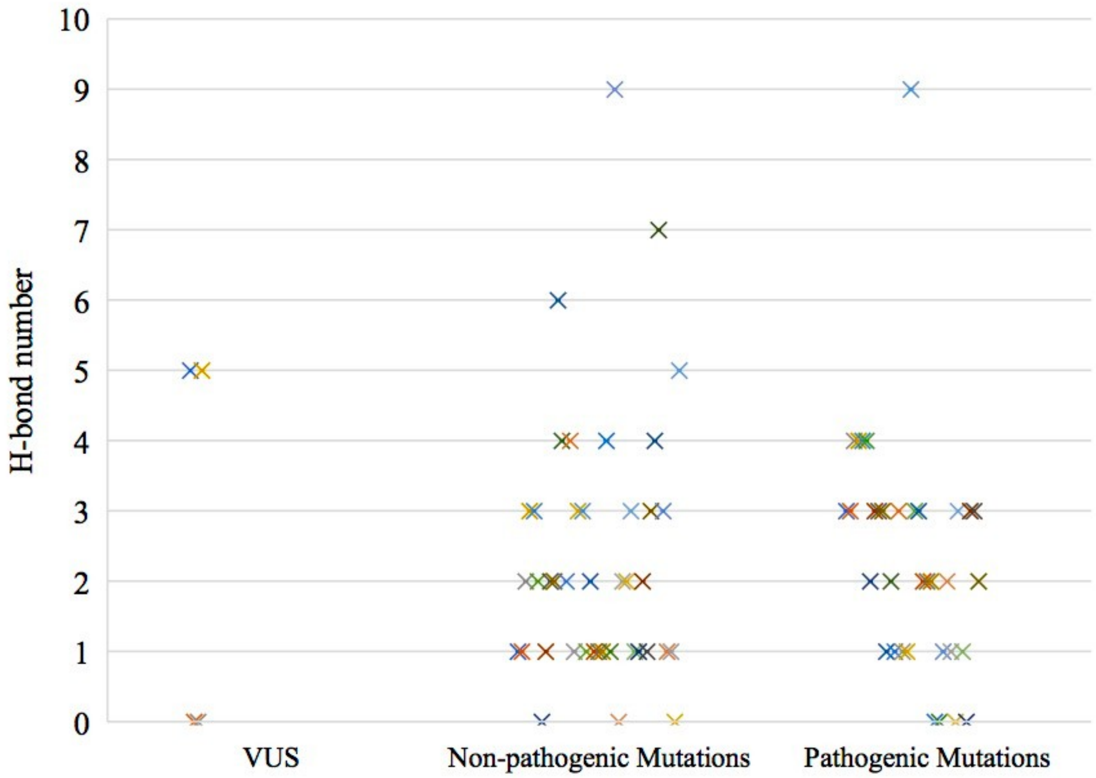

Figure S6B. H-Bond Numbers on MSH2 Wild-Type Residues. No significant difference between Non-pathogenic and pathogenic groups. Each cross present one data and no relationships between the colors.

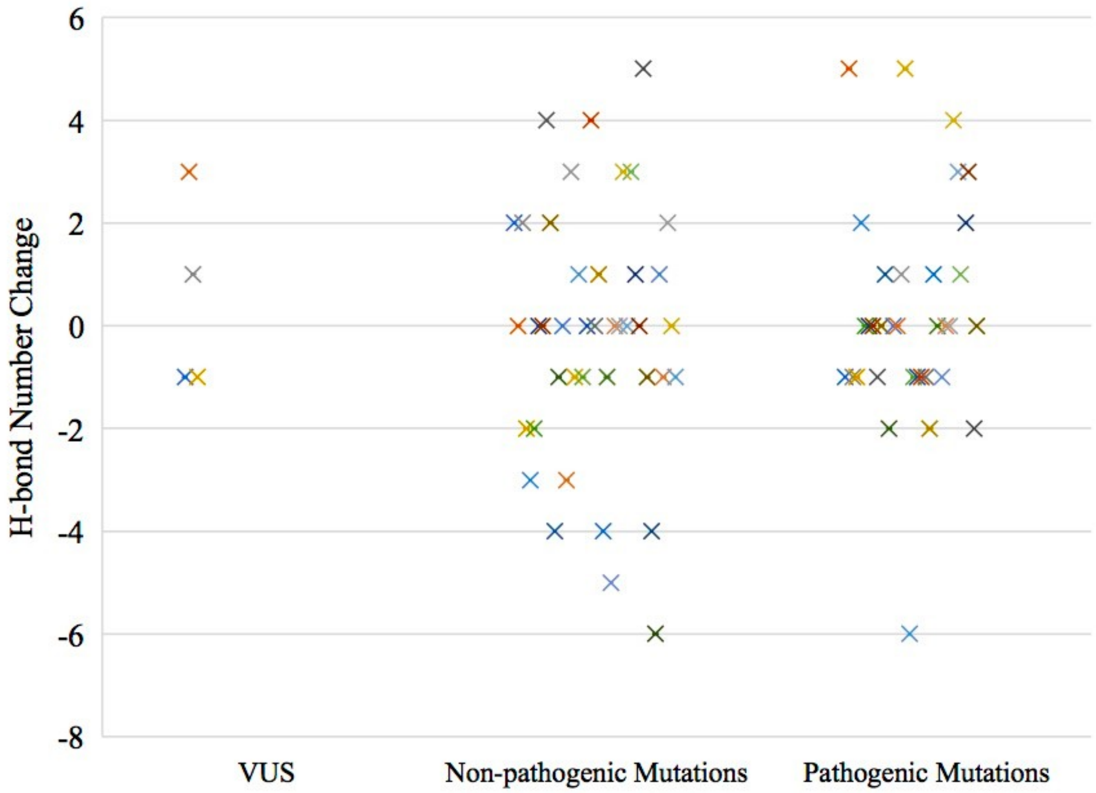

Figure S6C. The H-Bond Number Changes upon MSH2 Missense Mutations. No significant difference between Non-pathogenic and pathogenic groups. Each cross present one data and no relationships between the colors.

Supplementary materials: Tables

Table S1. Biological and Physicochemical Characteristics of Non-pathogenic Mutations (Selection 1)

Mutations	Folding ΔΔG	EC score	RMSD_ AVG	RMSF_WT	SIFT	Polyphen
p.Phe23Leu	-0.772	0.562	12.848	5.199	tolerated	benign
p.Ile169Val	-0.897	0.466	8.421	2.958	tolerated	benign
p.Gln419Lys	-0.210	0.658	8.655	2.029	tolerated	benign
p.Thr564Ala	-0.655	0.644	7.384	2.919	tolerated	benign
p.Gln629Arg	0.205	0.644	9.808	1.653	tolerated	benign
p.Ala2Thr	-0.963	0.795	6.189	7.404	deleterious	probably damaging
p.Thr8Met	-0.327	0.521	5.257	5.576	tolerated	benign
p.Met26Leu	-0.592	0.685	6.800	4.252	tolerated	benign
p.Arg96His	-1.106	0.836	6.038	2.675	deleterious	probably damaging
p.Val102Ile	-0.621	0.904	6.274	3.531	tolerated	probably damaging
p.Arg106Lys	-0.446	0.493	6.530	7.154	deleterious	benign
p.Asn127Ser	-0.864	0.904	5.205	2.765	deleterious	probably damaging
p.Asp167His	-0.786	0.877	6.655	1.902	deleterious	probably damaging
p.Glu198Gly	-1.973	0.890	6.013	1.459	deleterious	probably damaging
p.Gly322Asp	-1.455	0.767	6.883	5.551	tolerated	benign
p.Leu390Phe	-1.276	0.822	5.281	3.064	deleterious	benign
p.Ile577Thr	-1.336	0.836	5.462	3.194	deleterious	probably damaging
p.Val642Ile	-0.544	0.658	5.214	2.411	deleterious	probably damaging
p.Gly759Glu	-1.057	0.877	6.214	1.409	deleterious	probably damaging
p.Ala834Thr	-1.234	0.849	5.676	1.761	deleterious	probably damaging

Note: Folding ΔΔG: Folding free energy change; EC: Evolutionary Conservation; RMSD_AVG: RMSD average; RMSF_WT RMSF of wild-type residue.

Table S2. Biological and Physicochemical Characteristics of Non-Pathogenic Mutations (Selection 2)

Note:

Mutations	Folding ΔΔG	EC score	RMSD_AVG	RMSF_WT	SIFT	Polyphen
p.Pro5Gln	-1.122	0.726	10.570	6.077	deleterious	probably damaging
p.Pro5Leu	-0.813	0.726	7.728	6.077	deleterious	probably damaging
p.Thr32Ser	-0.963	0.795	12.848	2.344	tolerated	possibly damaging
p.Met141Val	0.256	0.726	9.836	2.258	tolerated	benign
p.Arg171Lys	-0.758	0.863	9.093	2.504	tolerated	possibly damaging
p.Lys228Glu	-0.247	0.904	8.955	2.482	deleterious	possibly damaging
p.Ser268Leu	0.748	0.904	12.601	2.179	deleterious	benign
p.Val273Ile	-0.344	0.822	9.775	1.876	tolerated	benign
p.Arg444Leu	0.135	0.589	8.222	2.162	tolerated	benign
p.His466Arg	-0.176	0.863	10.344	2.818	deleterious	possibly damaging
p.Ser479Arg	-0.684	0.658	7.051	2.692	tolerated	benign
p.Asp597Ala	-0.205	0.822	9.449	1.586	tolerated	possibly damaging
p.Ala640Ser	-1.816	0.548	9.388	2.062	tolerated	benign
p.Val655Ile	-0.472	0.671	11.693	1.216	tolerated	benign
p.Tyr656Cys	-0.853	0.247	7.041	1.185	tolerated	benign
p.Glu658Gly	-1.144	0.767	8.676	1.280	tolerated	benign
p.Met688Ile	-0.825	0.918	11.264	1.144	deleterious	probably damaging
p.Ile704Thr	-2.312	0.726	9.481	1.136	tolerated	probably damaging
p.His785Pro	0.532	0.877	9.679	1.561	deleterious	probably damaging
p.Glu809Lys	-0.400	0.562	9.317	6.698	tolerated	benign
p.Lys845Glu	0.4463	0.6027	11.9629	2.089	tolerated	benign

Folding ΔΔG: Folding free energy change; EC: Evolutionary Conservation; RMSD_AVG: RMSD average; RMSF_WT RMSF of wild-type residue.

Table S3. Biological and Physicochemical Characteristics of Pathogenic Mutations

Mutations	Folding ΔΔG	EC score	RMSD_AVG(Å)	RMSF_WT	SIFT	Polyphen
p.Val161Asp	-3.162	0.795	9.429	1.449	deleterious	probably damaging
p.Gly162Arg	-1.331	0.890	7.979	1.356	deleterious	probably damaging
p.Val163Gly	-2.498	0.890	12.151	1.275	deleterious	probably damaging
p.Val163Asp	-2.912	0.890	10.125	1.275	deleterious	possibly damaging
p.Gly164Arg	-1.143	0.863	9.430	1.336	deleterious	probably damaging
p.Gly164Glu	-1.799	0.863	10.845	1.336	deleterious	probably damaging
p.Leu173Arg	-1.466	0.767	10.343	1.621	deleterious	probably damaging
p.Leu187Arg	-2.217	0.890	8.404	1.253	deleterious	probably damaging
p.Leu187Pro	-2.184	0.890	9.473	1.253	deleterious	probably damaging
p.Cys199Arg	-1.439	0.890	15.646	1.460	deleterious	probably damaging
p.Val200Asp	-2.899	0.726	10.305	1.553	deleterious	benign
p.Leu310Pro	-2.183	0.904	9.221	1.133	deleterious	probably damaging
p.Cys333Tyr	-1.440	0.918	9.750	1.353	deleterious	probably damaging
p.Gly338Glu	-2.054	0.918	10.834	1.353	deleterious	probably damaging
p.Pro349Leu	-0.895	0.918	13.926	1.440	deleterious	probably damaging
p.Pro349Arg	-1.259	0.918	13.580	1.440	deleterious	probably damaging
p.Arg359Ser	-2.448	0.904	9.728	1.545	deleterious	possibly damaging
p.Leu440Pro	-2.813	0.890	10.409	1.887	deleterious	probably damaging
p.Met453Lys	-0.962	0.863	10.079	1.802	deleterious	benign
p.Ser554Cys	0.753	0.822	12.677	3.730	deleterious	probably damaging
p.Ser554Gly	-0.663	0.822	12.523	3.730	tolerated	benign
p.Ser554Thr	-0.725	0.822	11.123	3.730	tolerated	benign
p.Gly587Arg	-0.513	0.932	11.472	1.926	deleterious	probably damaging
p.Pro622Leu	1.154	0.932	12.334	1.318	deleterious	probably damaging
p.Ala636Pro	-1.023	0.616	7.804	1.388	tolerated	possibly damaging
p.His639Tyr	-0.925	0.932	7.359	1.766	deleterious	probably damaging
p.Gly669Val	-0.590	0.945	11.054	1.489	deleterious	probably damaging
p.Gly683Arg	-1.547	0.918	10.834	1.081	deleterious	probably damaging
p.Met688Arg	-1.530	0.918	9.010	1.144	deleterious	probably damaging
p.Gly692Arg	-1.472	0.397	12.480	1.425	deleterious	probably damaging
p.Pro696Leu	0.942	0.932	15.281	1.567	deleterious	probably damaging
p.Cys697Arg	-1.285	0.932	11.842	1.380	deleterious	probably damaging
p.Cys697Phe	-1.460	0.932	11.266	1.380	deleterious	probably damaging
p.Gly751Arg	-1.304	0.890	12.295	1.372	deleterious	probably damaging

Note: Folding ΔΔG: Folding free energy change; EC: Evolutionary Conservation; RMSD_AVG: RMSD average; RMSF_WT RMSF of wild-type residue.

Table S4. Biological and Physicochemical Characteristics of VUS

Mutations	p.Tyr43Cys	p.Ala272Val	p.Asn547Ser	p.Met592Val
Predictions	Non-Pathogenic	Pathogenic	Non-Pathogenic	Pathogenic
Folding Free Energy Change	-1.228	-0.788	-0.26	-1.286
EC score	0.767	0.877	0.863	0.575
RMSF_WT	4.559	1.966	5.725	1.876
SIFT	deleterious	deleterious	tolerated	tolerated
Polyphen	probably damaging	possibly damaging	possibly damaging	benign
Diagnosis	Intermediate bilateral stage III Size>1cm	high grade stage I BC size<1cm	intermediate, stage II A size<1cm	intermediate stage II A Size>1cm
BC mutations	BRCA2: c.4936_4939delGAAA	N/A	BRCA2: c.8791A>G	N/A
Other Biomarkers	ER(+);PR(+); Her2(-)	ER(-);PR(-); Her2(-)	ER(+);PR(-); Her2(+)	ER(+);PR(-); Her2(-)
Family History of BC/GI cancer	Yes/Yes	Yes/No	Yes/No	Yes/No

Note: Folding ΔΔG: Folding free energy change; RMSD_AVG: RMSD average; RMSF_WT RMSF of wild-type residue; BC: Breast Cancer; GI: Gastro-Intestinal Cancer.

Table S5. Clinical Characteristics of VUS Carriers

ID Number	B1	B2	B3	B4s
GGC Mutation Type	BRCA2: c.4936_4939delGAAA			
GGC VUS	MSH2: c.128A>G	MSH2 c.815C>T	MSH2: c.1640A>G; BRCA2: c.8791A>G	MSH2 c. 1774A>G
Tumor Size	>1cm	0-0.9 cm	0-0.9 cm	>1cm
ER Marker	(+)	(-)	(+)	(+)
PR Marker	(+)	(-)	(-)	(-)
Her2 Marker	(-)	(-)	(+)	(-)
TNBC	No	Yes	No	No
Surgery Intervention	Mastectomy	Lumpectomy	Lumpectomy	Lumpectomy
Stage	Stage IIIA	Stage I	Stage II B	Stage IIA
Family History of Breast ca	Yes	Yes	Yes	No
Family History of GI Ca	Yes	No	No	No
Tum Grade	Intermediate	High	Intermediate	Intermediate
Age	70-79	50-59	60-69	60-69
Ethnicity	White	White	Black	White
Age Onset	70	58	61	68
Menopause	Post menopausal	Post menopausal	Post menopausal	Post menopausal
Cancer site	Bilateral	Left	Bilateral	Left
Sentinel node	Sentinel Node Positive	Sentinel Node Negative	Sentinel Node Negative	Sentinel Node Negative
